# Editorial: In Memoriam of Professor Alessandro Moretta

**DOI:** 10.3389/fimmu.2020.00439

**Published:** 2020-03-13

**Authors:** Daniel Olive, Eric Vivier, Chiara Romagnani

**Affiliations:** ^1^INSERM UMR1068, CNRS UMR7258 Institut Paoli Calmettes, Marseille, France; ^2^Innate Pharma, Marseille, France; ^3^Deutsches Rheuma-Forschungszentrum (DRFZ), Berlin, Germany

**Keywords:** natural kill cell, transplanation, immune deficiency, infection - immunology, cancer biology

Prof. Alessandro Moretta was a major contributor to Frontiers in Immunology, particularly in the domain of NK cells and innate lymphocyte biology. We would like to honor his memory with a special tribute edition containing a collection of reviews covering the various periods of his scientific career, which was long, although sadly not as long as it might have been. The authors will try to connect his discoveries with the history of these cells to date. Their memories of these exciting times, full of enthusiasm, will be a useful resource for all readers. We would like the authors to share with the readers their discussions with him concerning the topic reviewed, and his view of the field, where possible.

These reviews will address the early years of discovery when Alessandro Moretta was a pioneer in the immune field, together with his brother Lorenzo and their collaborators, even before he began working on NK cells. By focusing on this period, we will highlight the tremendous enthusiasm surrounding these discoveries.

The activation of NK cells via the so-called “natural cytotoxicity receptors” (NCRs) 2B4, NTB-A and DNAM-1 will be highlighted by Vitale et al. and Pende et al. will address the discovery of NK-cell immunomodulation through the identification of killer cell Ig-like receptors (KIR) and the developments leading to its clinical use essentially in pediatric acute lymphoblastic leukemia.

Translational studies in the field of cancer will be presented by Pesce et al. for ovarian cancer and Leclerc et al., for lung carcinoma. Di Vito et al. will address the ontogeny of NK cells specifically post-allostem cell transplantation.

The primary immunodeficiency studies will be handled by Tabellini et al. as well as Zimmer for TAP transporter.

We all three think that our colleagues have really presented important insights that would have delighted Alessandro.

## Author Contributions

All authors listed have made a substantial, direct and intellectual contribution to the work, and approved it for publication.

### Conflict of Interest

EV is the CSO of Innate Pharma. DO is the cofounder of Imcheck Therapeutics. The remaining author declares that the research was conducted in the absence of any commercial or financial relationships that could be construed as a potential conflict of interest.

